# Grouping in object recognition: The role of a Gestalt law in letter identification

**DOI:** 10.1080/13546800802550134

**Published:** 2009-05-07

**Authors:** Denis G. Pelli, Najib J. Majaj, Noah Raizman, Christopher J. Christian, Edward Kim, Melanie C. Palomares

**Affiliations:** Psychology and Neural Science, New York University, New York, NY, USA

**Keywords:** Gestalt, Grouping, Contour integration, Good continuation, Letter identification, Object recognition, Features, Snake in the grass, Snake letters, Dot lattice

## Abstract

The Gestalt psychologists reported a set of laws describing how vision groups elements to recognize objects. The Gestalt laws “prescribe for us what we are to recognize ‘as one thing’” (Köhler, 1920). Were they right? Does object recognition involve grouping? Tests of the laws of grouping have been favourable, but mostly assessed only detection, not identification, of the compound object. The grouping of elements seen in the detection experiments with lattices and “snakes in the grass” is compelling, but falls far short of the vivid everyday experience of recognizing a familiar, meaningful, named thing, which mediates the ordinary identification of an object. Thus, after nearly a century, there is hardly any evidence that grouping plays a role in ordinary object recognition. To assess grouping in object recognition, we made letters out of grating patches and measured threshold contrast for identifying these letters in visual noise as a function of perturbation of grating orientation, phase, and offset. We define a new measure, “wiggle”, to characterize the degree to which these various perturbations violate the Gestalt law of good continuation. We find that efficiency for letter identification is inversely proportional to wiggle and is wholly determined by wiggle, independent of how the wiggle was produced. Thus the effects of three different kinds of shape perturbation on letter identifiability are predicted by a single measure of goodness of continuation. This shows that letter identification obeys the Gestalt law of good continuation and may be the first confirmation of the original Gestalt claim that object recognition involves grouping.

In what many take as the defining paper of the Gestalt movement in perception, Wertheimer (1923; translated in Ellis, 1938) made a bold claim, the laws of grouping, and set an ambitious goal, to understand object recognition. In that paper, “Laws of organization in perceptual forms”, he said: “I stand at the window and see a house, trees, sky.. . .. I gaze for a long time.. . . And I discover that part of a window sash and part of a bare branch together compose an *N*.” Wertheimer presented several “Gestalt” laws that describe how we group elements to see shape. The Gestalt laws “prescribe for us what we are to recognize ‘as one thing’” (Köhler, 1920; excerpts are translated in Ellis, 1938. We quote p. 168 [German]/p. 32 [English].). Nearly a century later, the laws have held up well. They are routinely mentioned and accepted. A few more have been added. None have been rejected. Wertheimer got the ball rolling by presenting compelling visual demonstrations of each law. For example, he presented regular lattices of dots with different spacings horizontally and vertically and showed that this spacing determined the perceived grouping into rows or columns (Figure 1). Many of us show Wertheimer's demonstration, unchanged, in our undergraduate classes in perception because it makes his point well. It shows a Gestalt law in action: Proximity promotes grouping. The grouping is assessed by the observer's binary preference (column vs. row). It is just as exciting today as in 1923 that simple principles can be demonstrated so easily, bringing us closer to understanding how we recognize objects. It was brilliant to play off horizontal versus vertical grouping, allowing simple printed demos to titrate the various laws of grouping against each other. It is a rich paradigm, still bearing fruit (Kubovy & van den Berg, 2008).

**Figure 1:**
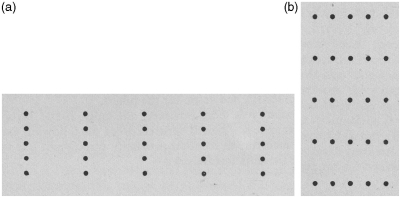
Dot lattices, reproduced from Wertheimer (1923), demonstrating grouping by proximity. Columns (left) or rows (right) are evident, but they are not familiar, meaningful, named things.

Palmer and Rock (1994, p. 30) said, “The Gestalt work on perceptual organization has been widely accepted as identifying crucial phenomena of perception, yet it has had curiously little impact on and integration with modern perceptual theory”. Wertheimer's dot lattice is exciting as a first step, but disappointing as an end point, if the goal is to understand object recognition. Everyone reports seeing grouping in the lattices, but seeing elements as a group is only a pale shadow of ordinary object recognition (Figures 1 and 2). Here we present experiments showing that grouping (specifically, good continuation) contributes to object recognition (identifying a letter). Before presenting our experiments, we draw the reader's attention to the surprising omission of object recognition in the large literature on grouping. Thus, this may be the first evidence that grouping contributes to object recognition.

**Figure 2:**
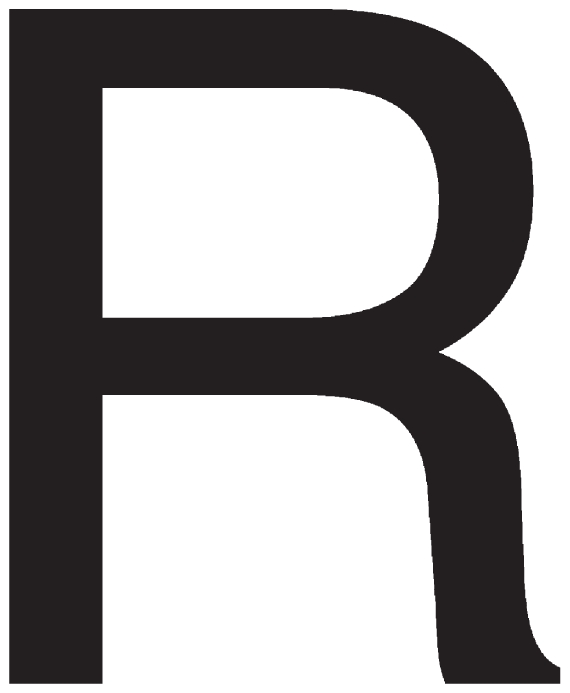
An R, demonstrating ordinary object recognition: a familiar, meaningful, named thing.

How can this be? Are there not innumerable examples in the literature of grouping influencing object recognition? What about the Gottschaldt embedded figures, and the many papers on figure–ground and a modal completion? Most of the studies of grouping, including those just mentioned, used binary discrimination tasks, which tend not to demand ordinary object recognition. Gottschaldt's (1926) embedded figures show that observers have great difficulty detecting a given familiar simple figure within a complex one; there is only one possible target, and it is a yes/no detection task. It is impressively difficult to find the target, but, even when one does succeed, this task does not seem to be an example of ordinary object recognition, which is quick, familiar, meaningful, and named. Recognition in the Gottschaldt task is familiar, but not quick.

## Grouping elements versus recognizing an object

We all spend our days recognizing objects, including words. This ordinary process of object recognition is a vivid rich experience. The object is typically a familiar thing. In recognizing it, we typically attach a short name and meaning, and we know its parts and what we can do with it (Gibson, 1979; Rosch, Mervis, Gray, Johnson, & Boyes-Braem, 1976; Tversky & Hemenway, 1984). That ordinary case corresponds, roughly, to *basic* object categorization (Rosch et al., 1976). Object recognition is vivid in the ordinary case, but becomes less and less vivid as the ordinary conditions (familiarity, name, meaning, basic category, one of many) are stripped away.

The essence of object recognition is the categorization. Thus the task assigned to the observer greatly affects the experience. We lack a hard and fast rule, but we can identify some important factors that affect whether the observer will see mere grouping of elements or quickly recognize a familiar, meaningful, named thing. Rosch et al. noted that superordinate and subordinate categories, larger and smaller than basic categories, have longer names and longer reaction times. Discrimination tasks (with two alternatives) seem less object oriented than identification tasks (with many alternatives). Object categories seem more evident in tasks that have a higher memory load (see “Categorical perception” below).

In psychophysics, we distinguish three tasks: detecting, discriminating, and identifying. In practice, the distinction boils down to the number of response categories allowed: two for detecting and discriminating, and more for identifying. We include detection as a special case of discrimination, because we can always think of the discrimination between A and B as detecting the difference, A minus B. So we concentrate on the difference between discriminating and identifying. Of course, discriminating between two possible objects is equivalent to identifying one of two possible objects. But, when identifying one of *n* objects, it turns out that the *n* = 2 case is very different from the rest, *n* > 2.

The familiar game of twenty questions is an effective way to probe someone's conception of an object. But, in that game, those binary yes/no questions are all different. When, in the typical psychophysical experiment, observers are asked to make the same binary perceptual discrimination again and again, the task, for the observer, seems less and less about objects and more and more about the raw stimulus experience, the sensation.

The *n* = 2 case is also special for the ideal observer. Ideal identification of one of *n* possible signals involves comparing the noisy stimulus with *n* templates (to calculate likelihood of the *n* hypotheses). (For treatment of the ideal identifier, see Van Trees, 1968, or Appendix A of Pelli, Burns, Farell, & Moore-Page, 2006.) However, as noted above, there is a shortcut when *n* = 2. Identifying one of two signals is equivalent to detecting the difference. The observer can compare the stimulus with the difference template and calculate the likelihood ratio of the two hypotheses and use that as the basis for the binary decision. This is mathematically equivalent and has the virtue of requiring less computation and, most important, requires memory of only one template. When *n* > 2, considering the differences among the signals is no longer attractive as there are at least as many differences (1 vs. 2, 2 vs. 3, 1 vs. 3) as signals.

By the way, there is nothing wrong with dots. Letters made of dots are still good letters. And let us not forget Johansson's (1973, 1975) point-light displays: “a few bright spots (5–12) in … motion evoke a vivid impression of a walking, running, etc., human being” (Johansson, 1976). “From only a few moving point lights, attached to the joints of an otherwise invisible moving actor, people readily perceive the underlying human figure, categorize the displayed action after viewing it for only fractions of a second, and can even perform subtle tasks such as gender recognition” (Beintema, Georg, & Lappe, 2006).

## No hard and fast rule

To our surprise, none of the factors that we have identified as important seem to be essential. Identifying a nameless squiggle (or Greeble) is less vivid than the ordinary case, but more vivid than mere grouping. Most binary discriminations fail to yield vivid objects, but the discrimination of certain object properties is much worse when the stimulus is not seen as an object. In these cases, good discrimination demands object recognition. For example, observers are much better at judging whether the barrel/hourglass illusory contour defined by four tilted corners is fat (barrel) or thin (hourglass) if the corners are perceived as four corners of one object rather than as four disconnected elements (Ringach & Shapley, 1996).

Our point is not merely semantic. We claim that mere grouping is not object recognition, but we do not know where to draw the line for what to accept as object recognition. We could relinquish that claim and accept mere grouping as a very weak form of object recognition, lacking essential qualities of ordinary object recognition. We insist that ordinary everyday object recognition is quick, familiar, meaningful, and named, and that most of these qualities are absent from most, if not all, of the existing tests of grouping.

## Categorical perception

One of the hallmarks of object recognition is categorical perception, whereby observers discriminate the same physical difference in a stimulus parameter much better if the difference crosses a category boundary, so that the two alternatives are perceived as different things. Consider a well-known example from speech perception. (For similar evidence of categorical perception in vision, see Goldstone, 1994.) A syllable sound can be synthesized with various voice onset times. The voice onset time can be set to any value on a continuous scale, but the synthesized sound is perceived as qualitatively different over that range—for example, ba, da, pa. The breaks between categories are at different voice onset times for native speakers of different languages. This categorization is demonstrated in a hard task, “ABX”, that requires the observer to retain three sounds in order to make a judgement. (The observer must say whether the stimulus was ABA or ABB, where A and B are sounds played one after another. A and B change randomly from trial to trial.) However, if the task is replaced by a binary discrimination of a single sound, A or B (which do not change within a block), the just-noticeable difference for voice onset time is reduced enormously and shows no correlation with the location of the category boundaries of the listener's language (Carney, Widin, & Viemeister, 1977).

The easy binary discrimination with light memory load seems to assess early sensory limits. The hard ABX task is also binary (identifying X as A or B) but has a high memory load and exhibits category boundaries like those observed in perception of speech, in which the memory load may be similarly high. It seems that we can judge a single sensory impression without categorizing coarsely, but when forced to compare several impressions we rely on object-based memories that are coarsely categorized.

## Snake in the grass

**Figure 3:**
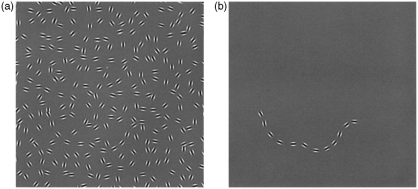
A snake (left) and a snake in the grass (right). From Field, Hayes, and Hess (1993). “In this example each successive element differs in orientation by ±30 deg and for this difference in orientation the string of aligned elements is easily detected.” We detect the “snake” but it is not a familiar, meaningful, named thing.

Subsequent investigators refined Wertheimer's dot lattice technique, studying factors that bias the observer to report the elements as grouped in one way or the other (Epstein, 1988; Hochberg & Peterson, 1987; Kanizsa, 1976; Kubovy, Holcombe, & Wagemans, 1998; Peterson & Gibson, 1994; Rock & Palmer, 1990). Field, Hayes, and Hess (1993) updated Wertheimer's paradigm, asking observers to detect a contour (a curvilinear grouping) in a field of randomly perturbed elements (gratings). This task is affectionately called detecting a “snake in the grass” (Figure 3). Elements (gratings) are placed along an invisible path. Proximity and good continuation among the elements increase the probability that the observer will see the elements as a group and thus detect the path. It is now well established that observers are less likely to detect contours that violate the Gestalt laws of good continuation (Beck, Rosenfeld, & Ivry, 1989; Brunswik & Kamiya, 1953; Dakin & Hess, 1998; Kovacs, 1996; Kovacs & Julesz, 1994; McIlhagga & Mullen, 1996). Geisler et al. measured the edge co-occurrence statistics of natural images and showed that a grouping model based on these statistics predicts the observer's contour-detection performance (Elder & Goldberg, 2002; Geisler, Perry, Super, & Gallogly, 2001). However, all this only addresses the observer's ability to detect (not identify) a contour. When the contour represents a recognizable shape, does grouping help identification, as Wertheimer claimed? Detection is a binary discrimination and, we argued above, does not typically result in object recognition. Just detecting (noting a difference) shows a role for grouping in perception, but falls short of showing a role in object recognition.

## Letters

Letter identification is mediated by feature detection (Pelli et al., 2006). The features are simple and detected independently. The feature detectors mediating letter identification are the same well-known spatial frequency channels as those that mediate grating detection (Majaj, Liang, Martelli, Berger, & Pelli, 2003; Majaj, Pelli, Kurshan, & Palomares, 2002; Solomon & Pelli, 1994). Despite reading a billion letters over a lifetime, people still recognize letters (and words) by detecting many simple features rather than detecting each letter (or word) as a whole, which would be much more efficient (Geisler & Murray, 2003; Pelli et al., 2006; Pelli, Farell, & Moore, 2003).

We argue above that the binary discrimination tasks typically do not demand object recognition, and that the observer may not be doing it. Requiring the observer to identify is important. But it is not necessary to bring the whole world into the lab to get observers to do ordinary object recognition. The humble task of identifying letters is enough. It is quick, familiar, meaningful, named recognition (Figure 2).

## Snake letters

**Figure 4:**
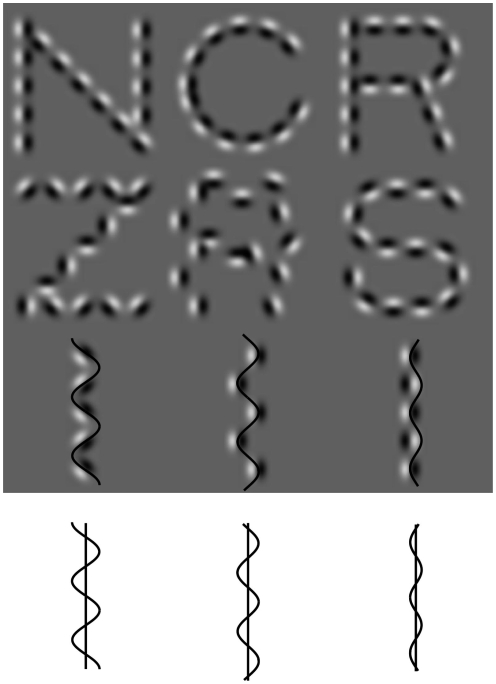
Measuring wiggle. The first row shows three unperturbed letters: The gratings are collinear with the path of the letter. The second row shows a sample letter for each of our three perturbations: orientation (Z), offset (R), and phase (S). In the third row, we use a straight stroke path and fit a sinusoid tangent to the white–black zero crossings nearest to the centre of each grating. White is always to the left of the sinusoid. Finally, in the bottom row, we measure the angle the sinusoid makes with its own axis.

To get back to Wertheimer's original goal of understanding object recognition, especially letter identification, we created letter-shaped contours and displayed them on a background of visual noise. Our alphabet, based on Sloan's, has 10 letters (see Method). This task allows us to directly measure efficiency of letter identification as a function of deviation from collinearity. Each standard (Sloan) letter is defined by the path a pen's stroke would follow in drawing it. In the standard condition, gratings are placed at regular intervals along the letter's (invisible) path, aligned with the path. We perturbed collinearity by rotating, offsetting, or phase shifting successive gratings, right or left alternately, relative to the path (Figure 4).

Figure 4 shows some perturbed letters. Note that the perturbation seems to bend the stroke, making it seem serpentine or wiggly. Inspired by this impression, we fitted a sinusoid, tangent to the white–black (not black–white) crossing nearest to the centre of the gratings. We define *wiggle* as the angle the sinusoid makes with its axis.

Each wiggled alphabet was created once and was then used unchanged through all training and testing.

The noise background was fresh (independent, identically distributed) on each presentation. The visual noise background swamps any additive intrinsic noise in the observer and makes the task an explicit computational problem, for which the optimal algorithm (maximum likelihood choice among the possible letters) may be solved mathematically and implemented as a computer program that represents the *ideal observer* (Appendix A of Pelli et al., 2006). We measured threshold contrast for 82% correct letter identification for both human and ideal observers. At threshold, we computed the contrast energy, integrated square of the contrast function over the signal area. The ratio of threshold energies, ideal over human, is called *efficiency* (Pelli & Farell, 1999).^1^ Efficiency strips away the intrinsic difficulty of the task to reveal a pure measure of human ability. See Pelli and Farell (1999) for a tutorial explaining how to measure efficiency.

Our paradigm is similar in some ways to the tumbling E test introduced by Levi, Sharma, and Klein (1997). Like our snake letters, their letters consisted of gabors. However, instead of adding a white noise background they perturbed the position of each gabor randomly on each presentation. Like us, they compare human and ideal thresholds to compute efficiency, but their manipulations did not assess the role of grouping, so their results are not relevant here.

More relevant is the tumbling C test of Saarinen and Levi (2001). They made a Landolt C (a perfect circle with a gap) out of gabors and presented the C at one of four orientations (90° apart), asking the observer to say which. They compared the threshold contrast for Cs made up of gabors that were all collinear with the C's path, or all orthogonal with the path, or each randomly collinear or orthogonal. Like us, they found that the orthogonal case resulted in higher thresholds than the collinear case. (The random case elevated threshold slightly more than the orthogonal case, but this difference was statistically significant for only one of the three observers, and in the group average.) They note in their abstract that their use of four-way identification was an advance on the prior work, which was all binary discrimination: “A number of previous studies have reported that integration of local information can aid ‘pop-out’ or enhance discrimination of figures embedded in distractors. Our study differs from the previous studies in that, rather than a figure–ground discrimination, our experiments measured contrast thresholds for shape identification” (Saarinen & Levi, 2001). While four-way identification is indeed an advance, it is our impression (confirmed by Levi, personal communication) that, at least subjectively, this particular task quickly reduces to detecting the gap and reporting its location, especially when near threshold. Thus, even though they were asking observers to identify four versions of a letter, it does not seem that the observers were doing ordinary object recognition.

**Figure 5:**
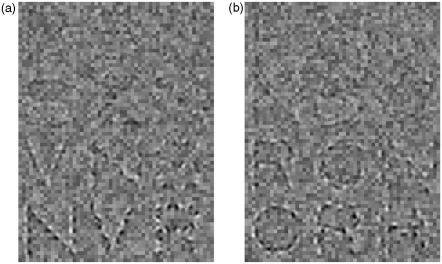
Letters in noise, demonstrating that good continuation is important for letter identification. For each letter chart, starting from the bottom, read up each column as far as you can. The height of the faintest identifiable letter is your contrast sensitivity for such letters. a. The orientation of the gratings relative to the letter stroke alternates ±0°, ±30°, or ±60° (left to right). b. The offset of the gratings from the letters stroke alternates ±0, ±2, or ±4 cycles (left to right). Letter contrast *L*_max_−*L*_background_) / *L*_background_ decreases by factors of √2 from 0.87 in the bottom row to 0.11 in the top row. The root mean square noise contrast is 0.15.

Figure 5 presents letter-chart versions of two of our three experiments, perturbing orientation (left panel) and offset (right panel). (We could not make a similar three-column chart for phase wiggle, because, as we defined it, there are only two strengths: on and off.) The perturbation increases from left to right. Letter contrast diminishes from bottom to top. For each column (perturbation) your efficiency is given by the highest (faintest) letter you can identify. Note the drop in efficiency as wiggle increases from left to right.

A “wiggle” is “a wavy line drawn by a pen, pencil, etc.” (*Oxford English Dictionary*, noun, 3). Others have measured sinusoidal curvature in order to study shape perception (e.g., Prins, Kingdom, & Hayes, 2007; Siddiqi, Kimia, Tannenbaum, & Zucker, 1999; Tyler, 1973; Wilson & Richards, 1989). Our treatment of wiggle is novel in applying one metric to three different kinds of perturbation, to test whether the effect of the various perturbations is mediated by this one parameter.

## Method

### Observers

There were two observers. C.J.C. was an undergraduate intern and is an author. A.S. was an undergraduate. Both observers had normal or corrected-to-normal acuity and were tested binocularly, with their correction, if any. Both gave written consent.

### Display

The stimulus consists of a letter added to a background of noise. The stimuli are created on a Power Macintosh using MATLAB and the Psychophysics Toolbox (Brainard, 1997; Pelli, 1997; http://psychtoolbox.org). The observer views a gamma-corrected computer monitor (Pelli & Zhang, 1991) from a distance of 100 cm. Each pixel subtends 0.019 deg. There are 74 pixels per inch. The frame rate is 75.5 Hz. The video attenuator drives just the green gun of the Apple 1700 Multiscan color monitor. The background luminance is set to the middle of the monitor's range, 16 cd/m^2^.

### Snake letters

In effect, we created a new font for each kind and degree of perturbation. This was done once. Each font, once made, never changed and was used unchanged throughout training and testing of both observers. (Here we are interested in how well an observer can perform, after adequate training with the font, in identifying a wiggly font. We are not studying how well observers can generalize from one font—and wiggle—to another.)

Each snake letter in a font is made from a letter path (based on Sloan), a perturbation rule (orientation, offset, or phase), a mark, and a mark spacing. The standard mark *m*(*x*, *y*) is a gabor, the product of a sine wave grating and a Gaussian envelope,

$$m(x,y)=\text{sin}(2\pi fx)\text{exp}\left(-\frac{x^{2}+y^{2}}{\lambda^{2}}\right)$$

where *m*(*x*, *y*) is a unit-contrast mark (vertical at the origin), *x* and *y* are horizontal and vertical positions in deg, *f* = 1 c/deg is spatial frequency, and λ = 0.3 deg is the space constant of its envelope. The mark interval (travel distance along the path from making a mark to making the next mark) is 0.91 deg.

Sloan is a special font designed by Louise Sloan for eye charts and contains only the 10 letters C D H K N O R S V Z. The shapes of the 10 Sloan characters are specified by the NAS-NRC Committee on Vision (NAS-NRC, 1980), based on Louise Sloan's design. Sloan is available from us for research purposes. We used MATLAB to create a simple Logo-like language in which we wrote a short computer program for each letter that describes its path as instructions to a “turtle” holding a pen—for example, activate pen, advance 1 unit, turn 90° to the right, deactivate pen, and so on [http://en.wikipedia.org/wiki/Logo_(programming_language)]. The imaginary turtle moved along the path. If the pen had ink, its stroke would draw the letter. However, to make a snake letter, the turtle instead made a mark at regular intervals during its pen-active travel along the path. The location and orientation of each mark are the same as those of the turtle, plus any perturbation. The sign of the perturbation was alternately positive and negative from mark to mark. A particular font had a particular kind of perturbation (orientation, offset, or phase) and a particular value (angle, displacement, or phase angle). The only nonzero phase perturbation we used was ±180. In this way the turtle walked the path of the letter and made many marks as it travelled. Each letter in the font consists of the sum of all the marks made during the turtle's walk along the letter's path. Each mark is a copy of the standard mark, moved and rotated to the turtle's current location and orientation, and possibly further rotated, offset, and phase shifted. The imaginary bounding box of the zero-thickness paths of the Sloan letters was 4 deg × 4 deg.

### Letters

All the fonts are rendered off screen (in computer memory) at a reduced scale. Independent Gaussian noise is added to each pixel. Then the image is expanded by pixel replication to its final size—each pixel growing to become a square check—and copied to the screen. The expansion is a doubling of size, horizontally and vertically. The experiments reported here present only one letter at a time, at fixation.

Ordinary reading presents many adjoining letters spanning a range of eccentricities. In that case, the number of letters that the observer can acquire in each glimpse is limited by “crowding” of the more peripheral letters (Pelli & Tillman, 2008; Pelli et al., 2007). However, there was no crowding in the experiments reported here.

### Noise

The noise is static, made up of square checks: 2 × 2 pixels. Each check is a luminance incrementor decrement, sampled independently from a zero-mean Gaussian distribution truncated at ±2 standard deviations. The power spectral density of a random checkerboard (with statistically independent check luminances) equals the product of the contrast power and the area of a noise check. The root mean square contrast of the noise is 0.15. The power spectral density of a random checkerboard (with stochastically independent check luminances) equals the product of contrast power and the area of a noise check. At a distance of 100 cm, a 2 × 2-pixel check subtends 0.041 deg so the power spectral density *N* is 0.15^2^ 0.041^2^ = 10^−4.42^ deg^2^. The noise covers the letter and extends 1 deg beyond its (invisible) bounding box.

### Training

For each observer, the results of the first 2,000 trials with each font were discarded before collecting the data reported here. This criterion is based on the finding that efficiency for identifying letters from a new alphabet initially grows rapidly but grows very slowly after 2,000 trials (Pelli et al., 2006).

### A trial: The identification task

On each trial, the observer is briefly shown a faint letter in visual noise and is then asked to select the letter from a display of all the letters in the alphabet. Each trial begins with the appearance of a fixation point on the grey background. The observer moves a mouse-controlled cursor to the fixation point and clicks the mouse button to initiate the trial. The stimulus consists of a signal and zero-mean white Gaussian noise added to the steady uniform background. The signal is a snake letter, randomly selected from a given font (see “Snake letters” above). This static stimulus is displayed for 200 ms and disappears. After a 200-ms delay, the whole alphabet is displayed at 80% contrast at the same size as the letter in the stimulus. The observer is asked to identify the signal letter by clicking a letter in the whole-alphabet display. After the observer responds, the correct letter is highlighted. A correct response is rewarded with a beep. The alphabet then disappears, and the fixation point reappears.

The signal letter and the whole-alphabet display are in the same font, and the font is the same for every trial in a block.

### A block: Threshold

The Michelson contrast *c* of a letter is (*L*_max_ − *L*_min_)/(*L*_max_ + *L*_min_). Threshold contrast is measured by 40-trial blocks of the modified Quest staircase procedure (King-Smith, Grigsby, Vingrys, Benes, & Supowit, 1994; Watson & Pelli, 1983) using a threshold criterion of 82% correct and a β of 3.5. For identification, the guessing rate γ is the reciprocal of the number of letters in the alphabet, since we presented all letters equally often. For experienced observers the proportion correct at high contrast is nearly 1, so we set the lapsing (or “finger error”) rate δ to 0.01. Threshold energy *E* for the alphabet is the average letter energy (across all the letters) at the threshold contrast for the alphabet. The reported efficiencies are based on the average log contrast threshold estimate from three 40-trial blocks.

Readers more accustomed to thinking about contrast should note that efficiency and energy are proportional to squared contrast.

### Efficiency

Tanner and Birdsall (1958) introduced the notion of comparing human and ideal thresholds to compute *efficiency*, η = *E*_ideal_/*E*. Here, *E* is the human observer's letter threshold energy measured in the presence of display noise with power spectral density *N*. *E*_ideal_ is the threshold for the ideal observer. Pelli and Farell (1999) point out several advantages to instead computing *high-noise efficiency*,

$$\eta^{*}=\frac{E_{\text{ideal}}}{E-E_{0}}.$$

*E*_0_ is the letter threshold energy for the human observer, measured with zero display noise. η* counts only the extra energy needed to overcome the display noise, discounting the energy needed to see the signal on a blank screen. The distinction between the two efficiencies, η* and η*—that is, the correction for the zero-noise threshold *E*_0_—becomes insignificant when the display noise is sufficiently strong to greatly elevate threshold, *E* > > *E*_0_. Since this was true for most of the efficiencies reported here, we just say “efficiency”, though it was always computed by Equation 2.

The ideal observer performs the same task as the human—identifying letters in noise—and we measure its threshold in the same way: On each trial the ideal-observer computer program receives a noisy stimulus and returns an identification response, which is scored as right or wrong. The mathematical description of the computation performed by the ideal observer is given by the theory of signal detectability for identifying one of many known signals in white noise (Van Trees, 1968). The ideal observer must decide from which of the 10 letters of the alphabet the letter-in-noise stimulus was most probably created.

The ideal observer is not intended as a model of the human observer. It merely provides a reference that allows us to place human performance on an absolute scale (Geisler, 1989). Human efficiency below 100% indicates a failure to fully utilize the available information. Finding a high human efficiency would rule out inefficient models. It is usually easy to impair an overly efficient model to match human efficiency, but difficult or impossible to salvage an inefficient model.

## Results

**Figure 6:**
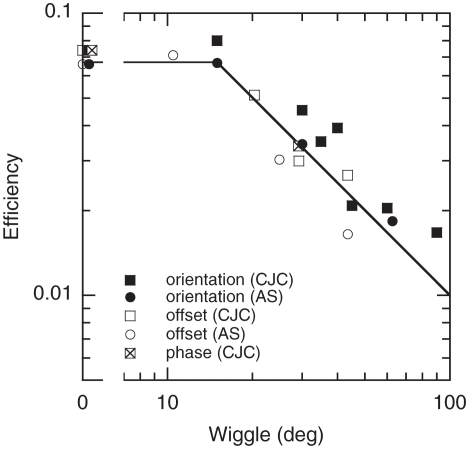
Efficiency as a function of wiggle of orientation (solid symbols), offset (open symbols), and phase (x-in-square symbol). The observers are C.J.C. (squares) and A.S. (circles). We measured threshold contrast energy (integrated square contrast) for letter identification as we perturbed grating orientation, offset, and relative phase. We implemented the ideal observer as a computer program and measured its threshold contrast energy for the same letter sets as those used for the human observer. The ratio of energy threshold of ideal to human is efficiency, an absolute scale that allows us to compare human performance across all our conditions (Pelli & Farell, 1999). Furthermore, it allows us to compare our new results to previous results for letters of various fonts, alphabets, and sizes. The bent line is the best fit of the clipped reciprocal η_0_(w) = η_0_ / min(1, w/w_0_), where *w* is wiggle, with two degrees of freedom, η_0_ = .074 and *w*_0_ = 15 deg, to minimize the fitting error (log η–log η),, where η is efficiency, and rms is root mean square.

Figure 6 plots efficiency as a function of wiggle for our three perturbations: orientation (solid symbols), offset (open symbols), and phase (x-in-square symbol). The three kinds of perturbation look quite different (Figure 4) but have the same effect on efficiency, all tracing out one curve. Efficiency is 8% at zero wiggle and falls, in inverse proportion to wiggle, for wiggles higher than 15°.

It is traditional for psychophysics papers to have a lot of data on few observers, which may seem strange to colleagues from other branches of psychology. In some studies in psychology the limited data that can be obtained from each observer are too variable to yield any conclusion. Averaging over many observers may overcome this, but at the cost of drawing conclusions that are valid for the average but may not be true of any individual. That is not the case here. In psychophysics, we are more concerned with the perceptual phenomena themselves than their incidence. Here, the data from each observer stand on their own, yielding valid conclusions for each observer. Given that our result is valid, independently, for each observer tested, we can say that it is valid for them (2 out of 2), and that this result must be fairly common in the population.^2^ Our data show both that this phenomenon is real for two people, and that it is quite common, with an incidence somewhere between 37% and 100%.

## Discussion

One simple explanation for the drop in efficiency with increasing wiggle might be that our human observers use internal letter templates that do not incorporate the wiggle exceeding 15°. The wiggle makes the letters different from the templates, making their identification harder. It is possible that observers used inaccurate templates, but it is not for lack of training. After only 2,000 trials, a novice observer's ability to identify letters of a foreign alphabet reaches the performance of life-long readers of that alphabet, and even an enormous amount of further practice provides relatively little further improvement (Geisler & Murray, 2003; Pelli et al., 2006). All results in Figure 6 are after at least 2,000 trials of practice with that perturbed set of letters and should thus be robust, little affected by the observer's prior exposure or further practice. If we trained without wiggle and tested with wiggle, then we would be assessing the observer's ability to generalize across wiggle.^3^ But in fact each experiment used the same wiggly alphabet for testing and training, so we are measuring the observer's ability to identify a wiggly alphabet, and the results show that more wiggle (poorer continuation) makes identification harder. More precisely, wiggle raises human threshold, but has no effect on the threshold of the ideal observer.

Why is the ideal immune to wiggle? It is proven in Appendix A of Pelli et al. (2006) that the threshold for the ideal observer depends only on the covariance among the *m* possible signals and the level of white noise (Equations A.12 and A.24). The wiggle has practically no effect on the covariance between letters, so the ideal's threshold is practically unaffected by wiggle. The intuition is that the ideal observer compares the noisy stimulus with each of *m* templates and picks the most similar. For the ideal it does not matter whether the templates are wiggly or not.

Another simple explanation is that wiggle increases complexity, the number of features in the object. Pelli et al. (2006) found inverse proportionality between efficiency and perimetric complexity, which they suggested might be a plausible estimate of number of features. That idea seems plausible here too. By this interpretation, grouping reduces the number of features, which improves the efficiency.

Past work has explored integration of nonoverlapping gratings using two experimental paradigms: contour integration and masking. Some scientists extended the paradigm of the early Gestalt psychologists, exploring the role of the Gestalt laws in the detection of objects (contours) made of gratings—that is, seeing a “snake in the grass”. In the masking paradigm, scientists studied how visibility of a single grating is affected by flanking gratings. We wondered how results with our “snake” letters would compare to existing results of the masking paradigm. We reexamined their data using our scales. Field et al. (1993) measured the probability of detecting contours consisting of relatively aligned gratings in a field of randomly oriented gratings. They found that proportion correct drops with increasing angle between successive gratings along the contour. We calculated the wiggle of their contours and converted proportion correct to (*d*′)^2^, which is proportional to efficiency. Log-log regression of (*d*′)^2^ versus wiggle reveals log-log slopes of −1.2 (*r* = .98) and −1.1 (*r* = .97) for their two observers, which agrees with the slope of −1 we fitted to our results. Solomon et al. measured the effect of nonoverlapping flanking gratings on the detection of a single grating, finding that the threshold contrast is a factor of √2 lower if the flanks are in phase with the target than if they are out of phase (Polat & Sagi, 1993; Solomon, Watson, & Morgan 1999). This is consistent with the difference in efficiency between our in- and out-of-phase letters.^4^

## CONCLUSION

Our experiments show that wiggle, a measure of sinusoidal curvature, characterizes the effect of good continuation on letter identification, independent of the kind of perturbation. This shows that this Gestalt law of grouping plays an important role in letter identification. This may be the first evidence that a Gestalt law of grouping plays a role in ordinary object recognition: quick, familiar, meaningful, and named.

## Footnotes

1 To be precise, we plot high-noise efficiency η^+^ = *E*_ideal_/(*E* − *E*_0_), where *E*_ideal_ and E are the ideal and human thresholds in noise, and *E*_0_ is the human threshold without noise, but the *E*_0_ correction is negligible at high noise levels (Pelli & Farell, 1999).

2 To be precise, if one takes two independent samples from a population of any size and discovers that both samples have a certain property, then one may conclude, with 95% confidence, that at least 37% of the population has that property. The webpage http://www.causascientia.org/math_stat/ProportionCI.html calculates the binomial confidence interval.

3 Our ability to read many fonts and handwritings suggests that we generalize well (Edelman, Flash, & Ullman, 1990).

4 The ideal observer's threshold is unaffected by our perturbation, so the factor of 2 difference in human efficiency represents a factor of 2 in threshold energy, which is a factor of √2 in threshold contrast.
